# Bedside HST and SVINT as Clinical Indicators Associated with Caloric DP and UW: A Retrospective Exploratory Study

**DOI:** 10.3390/audiolres16050147

**Published:** 2026-09-29

**Authors:** Mauro Gufoni, Giampiero Neri

**Affiliations:** 1 Ear Nose and Throat Unit, Ospedali Riuniti di Livorno, 57124 Livorno, Italy; mgufoni@alice.it; 2 Neurosciences Imaging and Clinical Sciences Department, University of Chieti-Pescara, 66100 Chieti, Italy

**Keywords:** acute unilateral vestibulopathy, unilateral weakness, directional preponderance, SVINT, head-shaking test

## Abstract

**Background:** Caloric testing provides quantitative measures of unilateral weakness (UW) and directional preponderance (DP). By contrast, skull vibration-induced nystagmus (SVINT) and the head-shaking test (HST) are bedside procedures that use different stimuli and operate within distinct temporal response domains. **Objectives:** This study was designed as a retrospective exploratory analysis of associations between categorical SVINT findings and caloric UW, and between categorical HST findings and caloric DP. **Methods:** We retrospectively evaluated 76 patients who underwent caloric testing, SVINT, and HST. Because the available records classified SVINT and HST only as present or absent, we restricted the analysis to associations between categorical findings and caloric classifications. **Results:** Pathological UW was associated with pathological SVINT, and pathological DP was associated with pathological HST. These findings are exploratory and do not demonstrate that the bedside procedures measure UW or DP, nor do they establish proportionality, physiological equivalence, interchangeability, or caloric-like quantitative information. **Conclusions:** SVINT and HST should therefore be interpreted as distinct and complementary bedside observations. Prospective studies with standardised quantitative eye-movement recordings are required to examine their temporal and physiological relationships with caloric responses.

## 1. Introduction

Skull vibration-induced nystagmus (SVINT) [1,2] and the head-shaking test (HST) [3,4] are accessible bedside procedures used to assess patients with vertigo. Caloric testing [5], despite causing less discomfort to patients [6], remains an important method for evaluating the relative function of the lateral semicircular canals and for calculating unilateral weakness (UW) and directional preponderance (DP) from quantitative slow-phase velocity measurements. However, the three procedures use different stimuli and probe different temporal aspects of vestibular processing.

Caloric testing produces a slow thermoconvective response that, through Jongkees’ formulas, shows unilateral weakness (UW) and directional preponderance (DP), [5] whereas SVINT is a vibration-bound response with nearly 90% sensitivity. It is not affected by compensation: it is still altered if the deficit persists [2,3,4,5,6,7]. The HST finally elicits the Head Shaking nystagmus (HSny), a dynamic after-response following head oscillation [2]. This HSny results from excitatory stimulation of the two hemisystems, whose algebraic sum occurs at the Velocity Storage level and is then downloaded at the end of the stimulation; if Velocity Storage is inefficient, an HSny cannot be evoked [8,9,10,11]. Their clinical findings should therefore not be assumed to be equivalent or to interrogate the same physiological construct. Bedside tests may provide useful complementary information, but they cannot substitute for quantitative caloric assessment when clinically required.

This study was designed as a retrospective exploratory analysis of associations between categorical SVINT findings and caloric UW, and between categorical HST findings and caloric DP. The hypothesis was deliberately limited to testing whether these bedside observations were associated with the corresponding caloric classifications in this sample. Because the records did not contain quantitative eye-movement measurements, the study was not designed to test proportionality, physiological equivalence, interchangeability, or temporal correspondence.

The primary objective was to estimate these prespecified categorical associations and to compare them with the corresponding cross-associations (UW–HST and DP–SVINT). The secondary objective was descriptive: to characterize the strength of categorical association and exploratory agreement without assigning a physiological identity to any test pair.

## 2. Materials and Methods

We reviewed the medical records of 76 consecutive patients evaluated in an audiovestibology unit for acute or stabilized unilateral vestibular deficits. All patients underwent caloric testing, SVINT, and HST as part of the clinical assessment. The study used existing clinical records and was conducted in accordance with applicable national legislation and institutional requirements.

All patients underwent history taking, audiometry, and vestibular examination, including assessment of spontaneous and evoked nystagmus using ocular and cephalic maneuvers. SVINT was assessed using a commercially available vestibular vibrator (Euroclinic Vestibular Vibrator ED500 (EUROCLINIC, Medi-Care Solutions S.r.l., Imola, Italy); fundamental frequency 100 Hz). Stimulation was applied for approximately 10 s behind each ear and, when clinically indicated, at the vertex. We observed responses with video-oculoscopy; no digitally stored eye-movement recordings were available for quantitative analysis. In the source records, SVINT was classified as positive when persistent horizontal–torsional nystagmus was observed during stimulation on at least two stimulation sites, and the finding was repeatable. This binary classification did not encode slow-phase velocity, amplitude, duration, stimulus-onset or stimulus-offset behavior, post-stimulation response, or interobserver reproducibility.

We performed HST and the clinical head-impulse test according to routine clinical practice. Patients with spontaneous nystagmus underwent the STANDING protocol [12,13]. When we suspected a central disorder, we assessed saccadic and pursuit eye movements and performed brain MRI when clinically indicated.

After initial symptomatic treatment, we proposed vestibular rehabilitation when clinically appropriate, with periodic follow-up examinations.

We performed caloric testing using hot and cold water. Twelve patients were tested during the acute phase, and 64 were tested 7 days after symptom onset. Clinicians determined the timing of testing, in part based on the severity of neurovegetative symptoms. We used the maximum slow-phase velocity elicited by each irrigation to calculate UW and DP using the Jongkees formulas. In the source clinical protocol, we classified UW and DP as abnormal when the calculated value was ≥30%. This prespecified operational cutoff was used for this retrospective exploratory analysis; for DP, it is consistent with the <30% normality criterion reported by Sills et al. [5]. The available retrospective records did not permit reconstruction of all technical details concerning calibration, objective recording systems, or interobserver reproducibility.

Inclusion criteria:

– Consecutive patients who underwent caloric testing, SVINT, and HST.
– Availability of clinical records and consent for the use of clinical data for research purposes, according to the applicable institutional requirements.

Exclusion criteria:

– Otological conditions that prevented caloric testing.
– Known central nervous system disorders.
– Refusal to undergo one of the study procedures.
– Absence of the required consent for use of clinical data.

Seventy-six patients were included: 37 females and 39 males, aged 17–87 years (mean 57.8 years, standard deviation 16.4 years).

The primary comparisons were UW versus SVINT and DP versus HST. We also evaluated cross-associations (UW versus HST and DP versus SVINT). Because the available bedside findings were categorical, the analyses addressed association and categorical concordance rather than quantitative proportionality.

https://www.R-project.org/ Statistical analyses were performed using R (R Core Team, R Foundation for Statistical Computing, Vienna, Austria; RStudio 2025.05.0, Posit Software, PBC) with the vcd, epiR, and forestplot packages.

We used chi-square tests of independence for the 2 × 2 tables. We used the phi coefficient to describe the strength of association, and calculated odds ratios with 95% confidence intervals where available.

– Exploratory diagnostic accuracy measures were calculated using the caloric classification as the reference: sensitivity, specificity, positive predictive value, negative predictive value, and likelihood ratios. We used Cohen’s kappa as an exploratory measure of categorical agreement.
– No analysis of continuous response variables, regression, or proportionality was possible because quantitative SVINT and HST measurements were not available.

The variables were therefore analyzed as categorical outcomes only.

The four variables were classified as follows:

– UW: pathological (UW+) or normal (UW-).
– DP: pathological (DP+) or normal (DP-).
– SVINT: pathological/observable (SVINT+) or absent (SVINT-).
– HST: pathological/observable (HST+) or absent (HST-).

The primary objective was to evaluate whether the two prespecified bedside findings were associated with the corresponding caloric classifications in this retrospective sample.

## 3. Results

Table 1, Table 2, Table 3 and Table 4 summarize the results. All results refer to categorical classifications.

The prespecified associations were stronger than the cross-associations. Pathological UW was associated with pathological SVINT (phi = 0.80; *p* < 0.001; odds ratio approximately 96). Pathological DP was associated with pathological HST (phi = 0.68; *p* < 0.001; odds ratio approximately 52). The corresponding exploratory accuracy and agreement estimates describe categorical concordance only. The cross-associations were weaker: UW–HST (phi = 0.29; *p* approximately 0.01) and DP–SVINT (phi = 0.35; *p* approximately 0.003). These findings identify associations for further investigation but do not show that either bedside procedure measures, estimates, or substitutes for a caloric parameter.

The results are compatible with categorical association in this selected retrospective sample. They provide no evidence about quantitative correspondence, stimulus–response dynamics, or shared physiological mechanisms, because the bedside responses were recorded only as present or absent.

This study should be interpreted strictly as a retrospective analysis of categorical associations. The observed relationship between UW and SVINT does not mean that SVINT measures UW, and the relationship between DP and HST does not mean that HST measures DP. The statistical analyses quantify the organization of the observed 2 × 2 tables; they do not establish that the tests interrogate the same physiological construct.

The procedures occupy different temporal and stimulus domains. Caloric irrigation produces a relatively slow, sustained thermoconvective response, quantified by slow-phase eye velocity and summarized by Jongkees’ formulas. SVINT is stimulus-bound: contemporary physiological accounts describe an abrupt onset with vibration, maintained activity during stimulation, and an abrupt offset, with little or no after-nystagmus in many patients with complete unilateral vestibular loss. HST is a dynamic after-response following head oscillation and is influenced by the velocity-storage network, compensation, visual-vestibular interaction, stimulus parameters, and examination timing [3,8,14,15]. These distinctions prevent a one-to-one interpretation of the three procedures.

The distinction between the immediate per-stimulatory SVINT response and any post-stimulation response is particularly important. A binary clinical record cannot determine whether an observed response was phase-locked to vibration, persisted after stimulus offset, or changed over time. Recent literature emphasizes that vibration-induced responses may reveal vibration-sensitive vestibular imbalance and should be interpreted as a distinct probe rather than a surrogate for caloric, vHIT, VEMPs, rotatory, or velocity-storage testing [16,17].

Post-SVINT after-nystagmus also requires separate consideration. Reports in superior semicircular canal dehiscence and third-window disorders describe measurable after-nystagmus. In contrast, after-nystagmus is generally small or absent after short-duration mastoid vibration in many patients with total unilateral vestibular loss [18,19]. These observations reinforce the need to distinguish stimulus-bound SVINT from post-stimulation phenomena and to prospectively record slow-phase velocity, duration, onset, offset, and reproducibility. The present dataset cannot address these questions.

HST should likewise be regarded as a context-dependent dynamic after-response, not as a direct measure of DP. Velocity storage integrates inputs from vestibular, visual, otolithic, and proprioceptive systems and contributes to prolonged nystagmus and after-responses. Longitudinal work indicates that the intensity of head-shaking nystagmus may change during vestibular compensation [18,19,20]. A negative HST may therefore reflect the absence of a measurable after-response under the specific testing conditions and does not exclude previous or persistent unilateral vestibular loss. Conversely, a positive HST may be affected by compensation, adaptation, visual suppression, cerebellar modulation, stimulus parameters, and timing [8].

The association between SVINT and UW may reflect shared sensitivity to vestibular asymmetry, but this remains one possible explanation among several, including selection effects, testing timing, binary classification, and central compensation. The association between HST and DP may similarly reflect overlapping clinical contexts rather than identity of the measured signals. No causal or physiological equivalence claim can be made from the present data [21].

The present data do not permit any conclusion about proportionality between vibratory nystagmus intensity and UW, or between HST response intensity and DP, because response intensity was not quantitatively recorded.

SVINT and HST may provide complementary bedside information when interpreted alongside the clinical history and examination. They should not be regarded as replacements for caloric testing or as caloric-like quantitative tests [22,23,24,25].

Potential practical advantages of bedside testing include accessibility, shorter examination time, and avoidance of ear-canal irrigation. These potential advantages do not eliminate the need to respect the distinct stimulus conditions, temporal domains, and limitations of each procedure.

The cross-tabulation shown in Table 1 describes the relationship between caloric UW and SVINT; it does not represent clinical combinations, stages of vestibular compensation, or validated diagnostic rules. Any clinical interpretation of the observed combinations is hypothesis-generating and requires prospective validation.

Accordingly, the observed combinations in Table 1 should not be considered definitive equivalents of caloric UW or DP, nor should they be used to assign a specific stage of vestibular compensation.

Interpretation of the bedside findings in temporal domains.

The combined presence or absence of SVINT and HST may be clinically descriptive. However, it cannot be used to infer a shared physiological mechanism or a stage of vestibular compensation. SVINT is linked to the period of vibration stimulation, whereas the response after head oscillation defines HST. Any combined interpretation must depend on the clinical context and the exact stimulus and recording conditions.

Caloric testing provides quantitative estimates of UW and DP using maximum slow-phase velocities elicited by irrigations and the Jongkees formulas. UW describes asymmetry in caloric responses between the two ears, whereas DP describes directional asymmetry in the induced nystagmus. These caloric parameters do not match the binary bedside findings analyzed here.

- Unilateral weakness (UW), also called canal paresis or reduced vestibular response, is a finding in caloric testing in which one inner ear’s balance response is significantly weaker than the other. It typically indicates a peripheral vestibular disorder, particularly regarding the relative function of the lateral semicircular canals and the direction of the provoked nystagmus.
- Unilateral weakness (UW), also known as canal paresis or reduced vestibular response, is a caloric-test finding in which one ear’s response is weaker than the other. It typically reflects asymmetry in the relative function of the lateral semicircular canals.
- Directional preponderance (DP) is the normalised difference between the peak slow-phase velocities (SPVs) of nystagmus elicited by irrigations expected to produce right- and left-beating nystagmus. Reported normal limits for DP have ranged from 20% to 50%; Sills et al. (1977) [5] defined a DP below 30% as normal. DP alone does not determine whether peripheral labyrinthine disease is irritative or deficient, and it may change during vestibular compensation.

In this study, pathological UW was associated with pathological SVINT, and pathological DP was associated with pathological HST. These categorical associations are hypothesis-generating only. They cannot establish quantitative proportionality, physiological equivalence, interchangeability, or a common temporal-domain mechanism.

The retrospective design, absence of stored video-oculographic recordings, dichotomous bedside classifications, incomplete information on technical reproducibility, absence of blinded assessment and interobserver reliability, lack of longitudinal stratification by compensation stage, and lack of direct comparison with quantitative vHIT [26,27] or other multidomain vestibular measures are important limitations. The study also included patients who underwent all three procedures, which may introduce selection bias. Prospective studies should use standardized stimulus protocols, blinded and repeated assessments, quantitative eye-movement recording, explicit separation of per-stimulatory and post-stimulatory responses, and direct comparison with caloric, vHIT, VEMPs, and appropriate measures of dynamic compensation [28].

## 4. Conclusions

In this retrospective exploratory study, categorical SVINT and HST findings were associated with caloric UW and DP, respectively.

The associations were stronger for UW–SVINT and DP–HST than for the cross-associations. Because SVINT and HST were assessed only as present or absent, the study does not demonstrate quantitative correspondence, proportionality, physiological equivalence, interchangeability, or shared physiological identity with caloric testing.

SVINT and HST may provide complementary bedside observations, but they should not be considered substitutes for quantitative caloric assessment or interpreted as caloric-like measures. Prospective studies using standardized quantitative recordings and a temporal-domain framework are required.

## Figures and Tables

**Table 1:** Comparison of caloric UW and SVINT. The association was strong (phi = 0.80; odds ratio approximately 96; chi-square approximately 48.6; *p* < 0.001). Exploratory sensitivity was 85.4%, specificity 94.3%, and Cohen’s kappa approximately 0.79.

	SVINT+	SVINT-	TOTAL
UW+	35	6	41
UW-	2	33	35
T	37	39	76

**Table 2:** Comparison of caloric DP and HST. The association was strong (phi = 0.68; odds ratio approximately 52; chi-square approximately 35; *p* < 0.001). Exploratory sensitivity was 84.6%, specificity 90.5%, and Cohen’s kappa approximately 0.67.

	HST+	HST-	TOTAL
DP+	11	2	13
DP-	6	57	63
T	17	59	76

**Table 3:** Comparison of caloric UW and HST. The association was weaker than the prespecified UW–SVINT association (phi = 0.29; odds ratio approximately 4.5; *p* approximately 0.01).

	HST+	HST-	TOTAL
UW+	15	26	41
UW-	4	31	35
T	19	57	76

**Table 4:** Comparison of caloric DP and SVINT. The association was weaker than the prespecified DP–HST association (phi = 0.35; odds ratio approximately 8.9; *p* approximately 0.003).

	SVINT+	SVINT-	TOTAL
DP+	11	2	13
DP-	24	39	63
T	35	41	76

## Data Availability

No new data were created or analyzed in this study. Data sharing is not applicable to this article.

## Abbreviations

The following abbreviations are used in this manuscript:

UW	Unilateral Weakness
SVINT	Skull Vibrational Nystagmus Test
SVINT	Skull Vibrational Nystagmus
HST	Head-Shaking Test
HSny	Post-HST Nystagmus
DP	Directional Preponderance
BFT	Bedside Functional Tests
PVL	Peripheral Vestibular Loss
